# Recent Developments in Organophosphorus Flame Retardants Containing P-C Bond and Their Applications

**DOI:** 10.3390/ma10070784

**Published:** 2017-07-11

**Authors:** Sophie Wendels, Thiebault Chavez, Martin Bonnet, Khalifah A. Salmeia, Sabyasachi Gaan

**Affiliations:** Additives and Chemistry Group, Advanced Fibers, Empa, Swiss Federal Laboratories for Materials Science and Technology, Lerchenfeldstrasse 5, 9014 St. Gallen, Switzerland; sophie.wendels@empa.ch (S.W.); thiebault.chavez@empa.ch (T.C.); martin.bonnet@empa.ch (M.B.)

**Keywords:** P-C bond, substitution, addition, Michaelis-Arbuzov reaction, Michael addition, flame retardant, organophosphorus compounds, transformation, TGA, Cone calorimetry, UL 94

## Abstract

Organophosphorus compounds containing P-C bonds are increasingly developed as flame retardant additives due to their excellent thermal and hydrolytic stability and ease of synthesis. The latest development (since 2010) in organophosphorus flame retardants containing P-C bonds summarized in this review. In this review, we have broadly classified such phosphorus compounds based on the carbon unit linked to the phosphorus atom i.e., could be a part of either an aliphatic or an aromatic unit. We have only considered those published literature where a P-C bond was created as a part of synthetic strategy to make either an intermediate or a final organophosphorus compound with an aim to use it as a flame retardant. General synthetic strategies to create P-C bonds are briefly discussed. Most popular synthetic strategies used for developing P-C containing phosphorus based flame retardants include Michael addition, Michaelis–Arbuzov, Friedels–Crafts and Grignard reactions. In general, most flame retardant derivatives discussed in this review have been prepared via a one- to two-step synthetic strategy with relatively high yields greater than 80%. Specific examples of P-C containing flame retardants synthesized via suitable synthetic strategy and their applications on various polymer systems are described in detail. Aliphatic phosphorus compounds being liquids or low melting solids are generally applied in polymers via coatings (cellulose) or are incorporated in the bulk of the polymers (epoxy, polyurethanes) during their polymerization as reactive or non-reactive additives. Substituents on the P atoms and the chemistry of the polymer matrix greatly influence the flame retardant behavior of these compounds (condensed phase vs. the gas phase). Recently, aromatic DOPO based phosphinate flame retardants have been developed with relatively higher thermal stabilities (>250 °C). Such compounds have potential as flame retardants for high temperature processable polymers such as polyesters and polyamides. A vast variety of P-C bond containing efficient flame retardants are being developed; however, further work in terms of their economical synthetic methods, detailed impact on mechanical properties and processability, long term durability and their toxicity and environmental impact is much needed for their potential commercial exploitations.

## 1. Introduction

P-C containing organophosphorus compounds find application as metal complex catalysts [1], reagents in organic chemistry [2,3], pesticides, insecticides, herbicides, medicine and biological systems [4], additives for polymers [5], surfactants, lubricants [6], surface treatments on metals [7], etc. In the past few decades, many review articles and book chapters regarding the creation of P-C bond and their potential applications have been published [6,8,9,10,11,12,13]. Organophosphorus compounds with P-C bonds are increasingly investigated as flame retardants due their high thermal and hydrolytic stability (P-C bond), ease of synthesis and suitability of processing at high temperature. One should pay attention to other properties like miscibility in the polymer matrix, reactivity with polymer or monomer, moisture absorption, tendency for blooming, toxicological properties, etc., which are very important and need to be considered before any commercial exploitation of these compounds can be realized. Unfortunately most published literature are academic and limited to aspects like synthesis, processability, flame retardancy and thermal properties of these compounds, thus it becomes very difficult to judge if any of these compounds discussed in this review can have any real commercial impact. The intention of this review is to summarize the work done in development flame retardants containing P-C bond without being critical of the shortcomings of the published literature.

P-C bonds are thermally quite stable and exhibit typical bond energies of ~272 kJ/mol [14]. Though the bond energies are lower than P-O (360 kJ/mol) and P-N (290 kJ/mol), the carbon substituents are not good leaving groups and thus resistant to possible nucleophilic attacks, which becomes relevant in high temperature polymer processing [15]. However, hydrolysis of P-C bonds can help in their biodegradation and reduce their possible harmful impact to the environment. Recent studies have provided insights into possible enzymatic hydrolysis of suitable P-C bonds [16,17,18]. Organophosphorus-based flame retardants are quite versatile in their flame retardant action and often exhibit both condensed and gas phase activity Depending on the substrate and phosphorus chemistry, there could be chemical interactions in the condensed phase at elevated temperatures, which lead to changes in the decomposition pathway of the polymer and possible formation of carbonaceous char residues on the surface of decomposing polymer, hence preventing its further oxidation. The char thus formed acts as a protective thermal barrier. In other instances, the phosphorus compounds and some of their decomposed products preferably volatilize from the polymer substrate when heated. These phosphorus species further decompose to release reactive phosphorus species, which then interact with the combustion intermediates in the gas phase as flame inhibitors [19,20]. In most cases, such interactions lead to recombination of the H and OH radicals and prevent their oxidation P-C bond containing phosphorus compounds like any organophosphorus flame retardants can exhibit either condensed phase or gas phase flame inhibition and is very specific to a polymer/flame retardant combination. For polymers (cellulose, wool), with relatively high concentration of hydroxyl or amino groups the phosphorus compounds primarily work in the condensed phase. In case of synthetic polymers containing oxygen and nitrogen atoms, catalytic hydrolysis of the ester or amide groups by phosphorus acids promotes an enhanced melt dripping and fast shrinkage from the flame. As far as olefin-based polymers are considered, the phosphorus compounds mainly act in the gas phase by recombining the key fuel species such as H and OH radicals and preventing their oxidation. Some minor physical effects due to volatilization of phosphorus compounds and dilution of the fuel can also occur [21].

In the following section, several analytical methods and techniques used by researchers to evaluate the thermal and fire properties of the new materials developed are mentioned. The choice of specific analytical technique depends mostly on the intended end use of the materials. TGA is generally employed to determine the thermal stability of new flame retardants and materials made from them. The degradation temperature of the flame retardant gives possible indication of its processing temperature. Higher char residue formed for the flame retardant polymer/formulation compared to the basis polymer indicates its possible condensed phase action. Similarly, higher char formation observed in other techniques such as PCFC and Cone calorimeter also indicate possible condensed based flame retardant action. Cone calorimeter and PCFC instrument is generally used to measure heat release of materials. Flame retardant action in condensed and gas phase leads in normally reduction in heat release rates. In cone calorimeter, one can also measure smoke and its composition. Flame retardants working in condensed phase usually leads to reduction in smoke toxicity. The ratio of CO/CO_2_ ratio as measured in the cone calorimeter indicates the activity of flame retardant in gas phase. Higher ratio for flame retardant materials indicates possible gas phase flame inhibition [22]. LOI is a common technique, which was used for evaluating the flame retardancy of textile materials and films. Materials exhibiting LOI values of 25% and greater are considered to self-extinguishing. Usually, inherently flame retardant materials exhibit LOI values greater than 30%.

Several small case fire tests like UL 94 and vertical burning tests are also commonly used to evaluate the flame retardancy of materials. In these tests, the reaction of materials to small fire either in vertical and horizontal orientation measured.

## 2. General Principles of P-C Bond Formation

The P-C bond formation has been reviewed in the past by various researchers [6,8,9,10,12,13,23,24,25,26,27,28,29,30]. Conventional synthetic strategies like Michael addition, Arbuzov rearrangement reactions, Friedel–Crafts reactions, radical addition reactions, Pudovik reactions are the most common strategy for P-C bond creation. In the last decade, there has been an increased focus on development of new synthetic methodologies for P-C bond formation. As a result, new novel and green methodologies [24,28] in the organic synthesis gave impetus to replace toxic and harmful phosphorus starting materials with more benign alternatives [31].

Due to the electronic nature of the phosphorus atom, the organophosphorus compounds can be classified into different chemical classes based on the coordination number and the oxidation state of the phosphorus atom [23,32]. Accordingly, the chemical reactivity of the organophosphorus compounds can be influenced by their structural geometry and valency of the phosphorus atom. For example, *H*-phosphonates with P(O)H bonds are commonly used as starting materials for P-C bond formation and thus their chemical reactions have been investigated in detail [6,9,12,24,25,29,30,31,33,34]. It is reported that presence of the P=O bond is the driving force for their reactions [23]. In general, the phosphorus center of the *H*-phosphonates is electrophilic and can be modified to be nucleophilic [35]. Moreover, the P-H bond of *H*-Phosphonates is considered acidic and can go through acid base reactions [36]. On the other hand, the *H*-phosphonates are quinquevalent, tetra-coordinated phosphorus compounds, which exist in equilibrium in solution with their corresponding tervalent, tri-coordinated species (phosphite ester). The phosphite esters have a lone electron pair on the phosphorus center and thus can react with various electrophiles [23]. When these phosphite esters are protonated or bear electron withdrawing groups, they react with nucleophiles. Based on the chemical reactivity of the organophosphorus compounds, diverse synthesis approaches of P-C bond formation have been explored and can be categorized as illustrated in Table 1, Table 2 and Table 3.

As seen in the table, there are numerous ways P-C bonds can be created and its potential academic research or industrial exploitations needs careful consideration. Flame retardant development is an applied research with a great potential for commercial exploitation and thus attention must be paid to the synthetic strategies for upscaling. In most cases, commercial availability of the starting chemicals and their cost determine their potential commercial exploitation. However, many other likely challenges can be encountered in industry, which hinders their industrial upscaling and utilities such as:

(1) Expensive synthesis approaches,
(2) Toxicity of the starting materials,
(3) Non-desirable wastes or by-product,
(4) Multiple purification steps,
(5) Difficulty handling of the chemical materials.

Currently only a few chemical reactions listed in Table 1, Table 2 and Table 3 are industrially exploited to manufacture flame retardants.

## 3. Flame Retardants with P-C_aliphatic_ Bonds

In the following sections, we have summarized examples of P-C_aliphatic_ bond containing organophosphorus compounds, which are classified based on the synthetic strategy for creation of P-C bond. For clarity, the specific P-C bonds that were created in the structure have been marked in bold.

### 3.1. Substitution Reactions

Substitution reactions are one of the most commonly used reactions used in organic chemistry and can be suitably exploited to create new bonds and eventually final products. Such reactions are commonly exploited commercially [53] and in research to create P-C bond containing useful flame retardants. In this section, we summarize development of organophosphorus compounds obtained by substitution reactions other than Grignard type reaction. The Grignard type reaction is described in detail later in the section.

**Figure 1:**
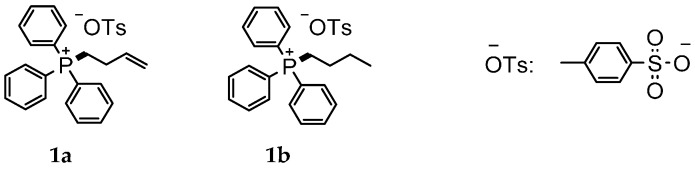
Chemical structures of **1a** and **1b** and their counter ion ^−^**OTs**.

The reaction of but-3-1-yl 4-methylbenzenesulfonate and butyl 4-methylbenzenesulfonate with triphenylphosphine afforded the phosphonium salts **1a** and **1b** respectively (Figure 1) [54]. The phosphonium salts **1a** and **1b** were obtained by precipitation in petroleum ether in 73% and 86% yield, respectively. Their thermal properties were studied using thermogravimetric analysis (TGA) under N_2_ atmosphere and the temperatures for 5% weight loss (T_d5%_) of 335.8 °C and 208.9 °C were observed for compounds **1a** and **1b**, respectively. They were then incorporated in polycarbonate (PC) composites at a concentration of 5% together with an anti-dripping agent. TGA was used to study the thermal stability of these composites. Cone calorimeter, UL94 and limiting oxygen index (LOI) tests were used to evaluate the flame retardant performance of the composites. It was found that the composite containing flame retardant with the alkene group (**1a)** exhibited a significant increase of LOI value (32.8%) compared to composite with compound **1b** (30.8%) and a lower peak heat release rate (PHRR). The LOI value of the neat PC is 25%. The char yield at 700 °C was found to be higher for both compounds (17 wt % and 15.6 wt % for compounds **1a** and **1b**, respectively) than for the virgin PC (13.9 wt %), which means that the two compounds improved the flame retardancy of the PC and act in the condensed phase. It was also noted for both compounds that 5% in the composite was enough to obtain UL 94 V-0 rating.

**Figure 2:**
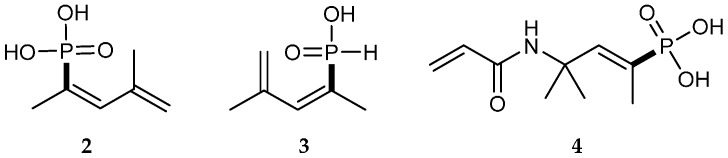
Chemical structures of **2**, **3** and **4**.

The conjugated dienes **2** and **3** (Figure 2) were synthesized by reaction of mesityl oxide with phosphorus acid and hypophosphorus acid, respectively [55,56]. It was described in the patent that these compounds can be used as flame retardants, potentially in the plastic industry or in textile coatings. The same researchers have also studied the synthesis of acrylamide derivative (Compound **4**, Figure 2) from phosphorus acid, mesityl oxide, acetic acid, acetic anhydride and phenothiazine and no additional fire performance of these compounds were reported [57].

**Figure 3:**
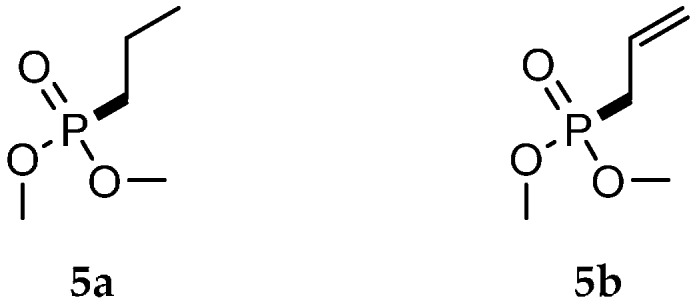
Chemical structures aliphatic phosphonates **5a** and **5b**.

Dimethylpropyl phosphonate (**5a**, Figure 3) and dimethylallyl phosphonate (**5b**, Figure 3) were synthesized by the reaction of dimethyl phosphite in presence of inorganic base with propylbromide and allylbromide, respectively. They were obtained as liquids in 92% (Compound **5a**) and 93% (Compound **5b**) yield. Both compounds were characterized using their boiling point: 105 °C at 23 mbar for **5a** and 104 °C at 24 mbar for **5b** [14]. They were incorporated in flexible polyurethane (PU) foams at concentrations of 1, 2, 5 and 10 wt % based on polyol content and their flame retardant performance were evaluated. The thermal stability and flame retardancy were investigated by TGA and PCFC, which showed that the flame retardant additives act predominantly in the gas phase. The flammability of PU foams were further investigated by LOI, UL 94 HB and BKZ-VB Swiss standard fire test. It was found at a 10 wt % (based on weight of polyol) flame retardant loading, all flame retarded PU passed the BKZ-VB compared to the virgin PU foam. Moreover, the flame retarded PU with compound **5a** showed a HF2 rating in the UL 94 HB test while the treated PU with compound **5b** showed HF1 rating. LOI values of the PU foams increased with increasing the flame retardant concentrations. The LOI values of treated PU foams with the flame retardant additives **5a** and **5b** were 22.8% and 23.9%, respectively. PCFC measurements of the flame retarded PU foams did not show a char residue which confirms their gas phase mode of action.

Compounds **5a** and **5b** (Figure 3) were also compared to a commonly used flame retardant tris(1-chloro-2-propyl) phosphate (TCPP) [58]. It was concluded from both studies that compounds with an allyl group are more efficient flame retardants for PU foams than compounds with alkyl groups.

**Figure 4:**
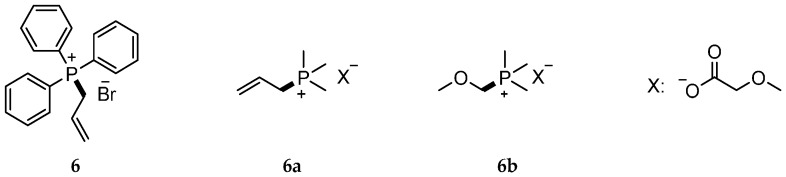
Chemical structures of phosphonium derivatives: **6**, **6a** and **6b**.

Allyltriphenylphosphonium bromide (Compound **6**, Figure 4) was synthesized from the reaction of triphenylphosphine and allyl bromide in dry toluene [59]. Subsequently, bentonite clay was modified with this phosphonium salt [60]. The surface modified bentonite clay was then used as a flame retardant additive (1, 2 and 3 wt %) for possible improvement in flame resistance and mechanical properties of polyester acrylate base UV-curable coatings. Tensile properties of films were evaluated from the stress–strain curve. The Young’s modulus, tensile strength and elongation at break of the films were determined. It was found that the addition of the modified clay increased the Young’s modulus but decreased the tensile strength. The elongation at break at first decreased but then increased with the increasing clay content. The coating became more brittle with addition of modified clay. The thermal properties and flammability properties were then investigated using TGA and LOI. The T_d10%_ of the neat coating was 373 °C while addition of clay first increased this value (the formulation with 1% modified clay showed a T_d10%_ of 387 °C), but then decreased to 361 °C for the formulation with modified clay content of 3 wt %. The char yield increased significantly from no char for the neat coating to 12% for the 3 wt % modified clay/coatings. The same trend was observed in the LOI measurements 17% and 22% for unmodified and 3 wt % clay/coatings. It was concluded from these results that the addition of flame retardant additive **6** to the clay had a significant impact on the mechanical as well as the flame retardancy of the polyester acrylate base UV-curable coatings acting dominantly in the condensed phase. All results were attributed to the crosslinking density of the allyl modified clay containing UV-cured coating.

Ionic liquids are known to be nonflammable and have recently generated a lot of attention as environment friendly reagents. Compounds **6a** and **6b** (Figure 4) are ionic liquids and were synthesized from trimethylphosphine, methoxyacetic acid and allyl chloride (**6a**) or chloromethyl methyl ether (**6b**) [61]. Associated with methoxyacetate as counter anion, these compounds are described as the optimal ionic liquids for the dissolution of cellulose; however, no real application data was presented in the paper.

**Figure 5:**
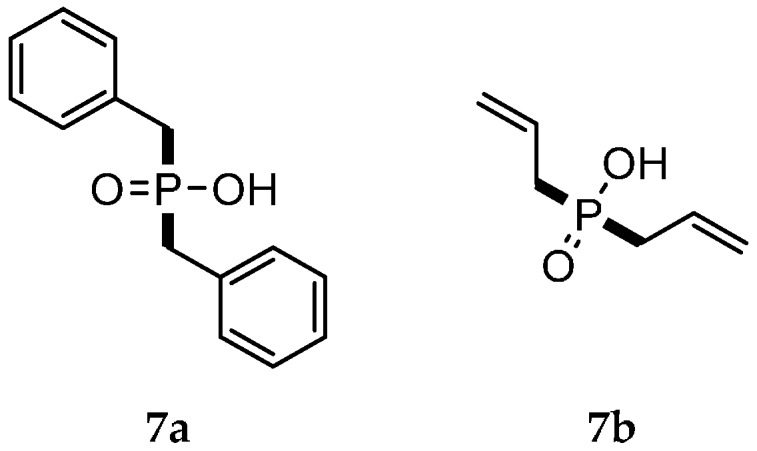
Chemical structure of **7a** and **7b**.

Recently, synthesis and application of different phosphinic acid derivatives has been reported [7]. Dibenzylphosphinic acid (**7a**, Figure 5) was synthesized from reaction of H_2_PO_2_NH_4_ with benzyl bromide; diallylphosphinic acid (**7b**, Figure 5) was synthesized from reaction of H_2_PO_2_NH_4_ with allyl bromide. These compounds were developed for application in epoxy resins for flame retardant and mechanical properties improvement. They were then treated with aluminium hydroxide to give aluminium–organophosphorus hybrids (AOPH) as follows: compound **7a** gave AOPH-NR and compound **7b** gave AOPH-C2. AOPH derivatives were then applied in epoxy resins to produce composites. LOI and TGA were performed for these composites and the analytical results showed that the AOPH-NR exhibits the best flame retardant properties: LOI for AOPH-NR was 28% and 23.6% for AOPH-C2. Moreover, the TGA analysis show that only AOPH-NR had an impact on the decomposition of the epoxy composites: the 5% and 50% decomposition temperatures decreased for AOPH-NR, whereas the other AOPH composites showed no real difference in thermal performance compared to the neat resin. TGA data indicated a slight increase in the char residue at 500 °C for both composites compared to the neat epoxy resin, showing a flame retardant mode of action in the condensed phase.

### 3.2. Addition Reactions

Addition reactions are also commonly used to create P-C bond containing flame retardants. The following section summarizes organophosphorus compounds obtained via addition reactions other than Michaelis–Arbuzov type reaction, which will be described in detail in the subsequent section.

**Figure 6:**
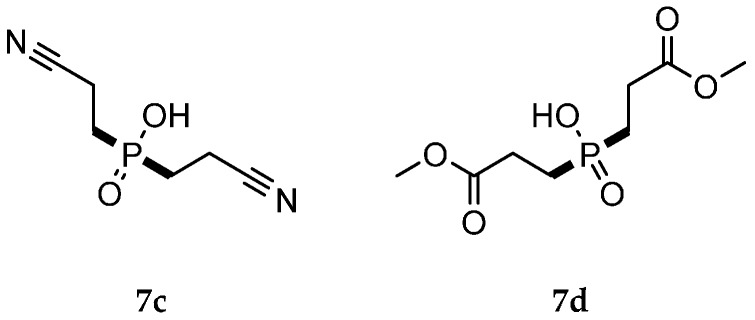
Phosphinic acid derivatives **7c** and **7d**.

Phosphinic acid derivatives bis(2-cyanoethyl)phosphinic acid (**7c**, Figure 6) was synthesized from reaction of H_2_PO_2_NH_4_ with allyl cyanide; and bis(3-methoxy-3-oxopropyl)phosphinic acid (**7d**, Figure 6) was synthesized from reaction of H_2_PO_2_NH_4_ with methyl acrylate [7]. These products were subsequently treated with aluminium hydroxide to give aluminium–organophosphorous hybrids (AOPH). The produced AOPH derivatives were also applied in epoxy resins.

**Figure 7:**
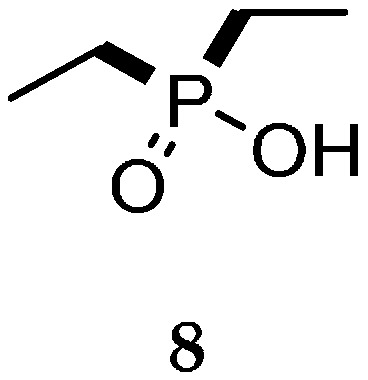
Chemical structure of **8**.

Diethylphosphinic acid (**8**, Figure 7) was obtained from a gas-liquid free-radical addition of sodium hypophosphite monohydrate and ethylene in acetic acid using benzoyl peroxide as initiator [62]. The thermal stability of compound **8** was investigated by TGA and the results showed two degradation peaks at 396 °C (small) and 424 °C (large), corresponding to the P-C bond breakage and combustion gas release, respectively. This compound was reported as a potential flame retardant; however, no further fire performance of this compound was reported.

**Figure 8:**
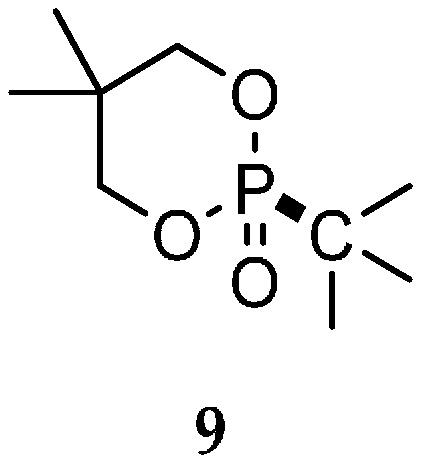
Cyclic *tert*-butyl phosphonate **9**.

The synthesis of a cyclic *tert*-butyl phosphonate (Compound **9**, Figure 8) is patented. Compound **9** was obtained by reaction of *tert*-butyl phosphonic dichloride and neopenthyl glycol [63]. This compound was claimed as a flame retardant due to the presence of phosphorus atom and can be used as an additive for polymer resin like polyphenylene ether. The Izod impact strength as well as the melt viscosity index at 200 °C were measured for a formulation with a composition of 85 wt % aromatic vinyl resin, 15 wt % polyphenylene ether resin and 20 wt % compound **9** of the final resin. The results showed 8.8 kgf.cm/cm Izod impact strength and a melt viscosity index of 7.9. In addition, this formulation also exhibited UL 94 V-0 rating.

**Figure 9:**
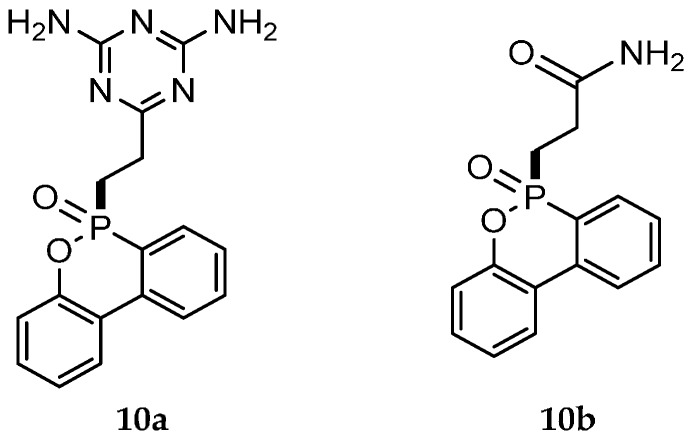
DOPO derivatives of **10a** and **10b** structures.

The triazine-based DOPO compound (**10a**, Figure 9) was synthesized using Michael addition reaction of DOPO with 2-vinyl-4,6-diamino-1,3,5-triazine using DBU as a catalyst [15]. The product was collected as a white solid power in 93% yield with a m.p. of ~257 °C. It was dried overnight and then used as a PA6 additive in co-extrusion. Its impact on flammability, thermal, mechanical and rheological properties for PA6 were studied and compared to a commercially available flame retardant Exolit^®^ OP 1230. PA6 formulations containing 17.1 wt % of compound **10a** and 16.8 wt % of Exolit^®^ OP 1230 were prepared. Detailed thermal, mechanical and morphological characterizations using TGA, DSC, Scanning Electron Microscope (SEM), tensile tests, hardness and Charpy impact strength measurements were performed for these formulations. Flammability measurements and mechanistic studies were performed using PCFC, UL 94, vertical burning test and direct insertion probe mass spectroscopy (DIP-MS). It was found that in N_2_ environment, neat PA6 showed a T_d5%_ of 388 °C and exhibited a one-step degradation process. In case of PA6 formulations, the T_d5%_ of the formulation decreased to 386 °C and 372 °C for Exolit^®^ OP 1230 and compound **10a** respectively. In air, the decomposition took place in three steps for all formulations and the T_d5%_ followed the same trend as in N_2_ environment. Thus, the addition of both flame retardants lowered the thermal stability of the PA6 polymer. Mechanical tests showed that the incorporation of compound **10a** in the PA6 formulation resulted in a more rigid and brittle material (higher Young’s modulus but an impact strength of 4.53 kJ/m^2^). Neat PA6 did not break in the Charpy test. PCFC measurements registered a PHRR of 738 W/g for PA6/**10a** formulation, followed by PA6 (590 W/g) and PA6/Exolit^®^ OP 1230 formulation (538 W/g). This high PHRR for PA6/**10a** formulation may be attributed to a catalytic decomposition of PA6 formed from the decomposition of the DOPO-derivative at high temperature. However, based on the THR, compound **10a** showed a similar behavior compared to Exolit^®^ OP 1230 (~27 kJ/g for both) and decreased the THR of the PA6 (28.5 kJ/g). The best UL 94 results were obtained for PA6 containing compound **10a** with a V-0 rating and afterflame times t_1_/t_2_ (time the flame stays after the first/the second flame) of 0 s/0 s respectively (2 s/3 s for Exolit^®^ OP 1230 and 2 s/0 s for the neat PA6). The char yield under N_2_ at 700 °C was found significantly higher for the PA6/**10a** formulation (2.4 wt %) compared to the PA6/Exolit^®^ OP 1230 formulation (0.9 wt %) and the neat PA6 (0.28 wt %). Based on all analytical data, it was concluded that compound **10a** exhibit both gas and condensed phase active flame retardancy for PA6, and can be considered as a suitable alternative to halogen and halogen free flame retardants commercially available for PA6 application.

The same group also synthesized DOPO-derivative **10b** (Figure 9) and compared its thermal and fire performances with compound **10a** [64]. Like in case of **10a**, **10b** was obtained using the synthetic procedure i.e., Michael addition of DOPO with acrylamide using 1,8-diazabicyclo(5.4.0)undec-7-ene (DBU) as a catalyst. It was obtained in a 75% yield and with m.p. ~ 170 °C. Both flame retardants were incorporated in rigid polyurethane foams (RPUF) with 1 wt % and 2 wt % phosphorus content and analyzed using UL 94 HB test, LOI, cone calorimetry, PCFC, TGA, DSC, SEM and Pyrolysis-Gas Chromatography Mass Spectroscopy (Py-GC-MS). Results showed that both flame retardants had HF-1 UL 94 HB rating while neat RPUF had a HBF rate. LOI values increased from 19.3% for the neat foam to 20.5% for PU foams containing compound **10b** and 20.9% for compound **10a**. Such increase in LOI are considered rather low compared to self-extinguishing systems which usually exhibit LOI > 25%. The cone calorimetry tests showed a significant decrease of the THR with the addition of flame retardant **10a** (23.1 MJ/m^2^) and compound **10b** (19.3 MJ/m^2^) compared to the neat PU foam (24.9 MJ/m^2^). TGA showed a slight decrease of the T_d10%_ for RPUF containing flame retardants **10a** and **10b** (300 °C for neat RPUF, 298 °C and 296 °C for RPUF with **10a** and **10b**, respectively). Evolved gas analysis, cone calorimetry and TGA experiments proved that DOPO derivatives-containing RPUF exhibit lower smoke and toxicant production and increased char residue, indicating the condensed phase mode of action.

### 3.3. Michaelis–Arbuzov Type Reactions and Rearrangement

**Scheme 1:**
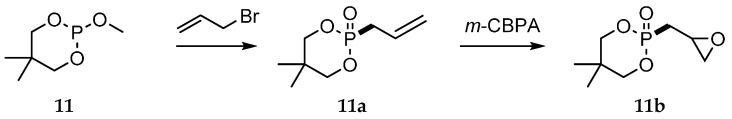
Chemical synthesis of compound **11b**.

Michaelis–Arbuzov reactions are one of the most common reactions used for the creation of phosphonate compounds. For example, Michaelis–Arbuzov reaction of 2-methoxy-5,5-dimethyl-1,3,2-dioxaphosphinane (**11**, Scheme 1) with allyl bromide produced 2-oxo-2-allyl-5,5-dimethyl-1,3,2-dioxaphosphinane (**11a**, Scheme 1). Epoxidation reaction of the later compound using *m*-CBPA, afforded the epoxy containing cyclic phosphonate **11b** [65]. Compound **11b** was reported as a white solid with 80% yield and a m.p. ~ 57–59 °C. It was then mixed with polyurethane (PU) at a 10 molar % in two different methods. The *Blend* method involved incorporation of the flame retardant as additive after the polymerization of the PU whereas the *Prep* method involved copolymerizing compound **11b** with PU. Pyrolysis combustion flow calorimetry (PCFC) test was used to evaluate the THR of the PU samples. It was found that the neat and the *Prep* PU had nearly the same THR (~22 kJ/g), which was lower than that for the *Blend* method (~25 kJ/g). The PCFC results confirmed that PU manufactured by both methods had no effect on THR of the PU. A possible explanation for this observation was attributed to the loss of compound **11b** during the final washing step in PU *Prep* method as well as to its weak incorporation in PU in the *Blend* method.

**Scheme 2:**
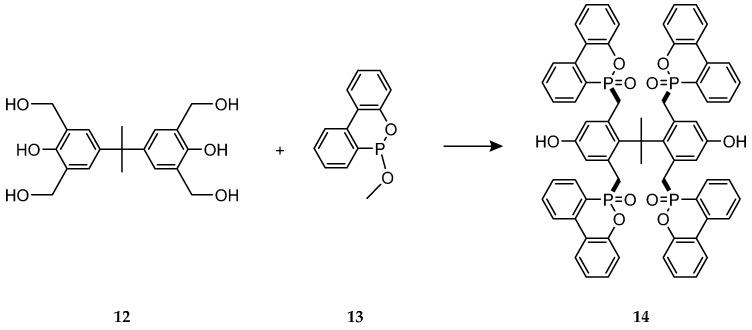
Synthetic route of compound **14**.

Compound **14** (Scheme 2) was synthesized from reaction of compound **12** (Scheme 2) with DOPO derivative **13** (Scheme 2), via Michaelis–Arbuzov rearrangement [66]. The synthesized phosphorous compounds were then used as flame retardants additives in epoxy resins.

**Figure 10:**
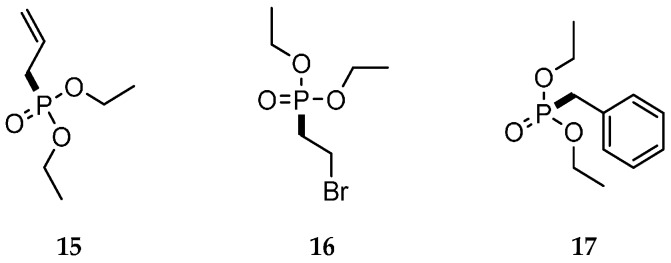
Flame retardant compounds **15**, **16** and **17**.

Diethyl allylphosphonate (**15**, Figure 10) has been synthesized from triethyl phosphite and allyl bromide and used in free radical precipitation polymerization in aqueous phase to produce poly(urethane-co-ester) (PMMD) copolymers [67].

The PMMD copolymers were synthesized from poly(ethylene glycol), methacrylate, methyl methacrylate and monomer **15** in different molar ratios 1:6:2 (PMMD-162) and 1:6:3 (PMMD-163). BMMD copolymers were blended with ABS to improve its flame retardancy. Improvements in flame retardancy have been observed using LOI and UL 94 tests. LOI values were 20.8% for 20 wt % PMMD-162 and 20.3% for 20 wt % PMMD-163, while neat ABS showed an LOI value of 17.8%. UL 94 tests also showed better fire results with increased amount of FR.

Diethyl bromoethylphosphonate (**16**, Figure 10) has been synthesized from 1,2-dibromoethane and triethyl phosphite to improve flame resistance of several polymers [68]. Compound **16** was used to synthesize other monomers for ROMP homopolymerization. Dimethyl benzylphosphonate (**17**) was synthesized from trimethyl phosphite and benzyl bromide and was applied in PU foam to improve the flame retardancy of PU foams. The structural influence of the organophosphorous compounds on its flame retardancy was also studied [14]. It was reported that applying compound **17** in PU foams improves the flame retardancy of PU foams as confirmed by LOI, BKZ-VB and UL 94 HB tests. The concentrations of compound **17** used in these experiments were 1, 2, 5 and 10%. LOI values for the formulations ranged from 21.2% for the lowest to 22.5% for the highest concentrate respectively. Burning velocity decreased from 5.9 mm/s for the least concentration of additive to 4.1 mm/s for the highest concentration. UL 94 HB tests confirmed HBF rating for the PU foam with 1% to 5% concentration of the flame retardant additive **17** and it improved to an HF2 rating for 10% concentration.

**Figure 11:**
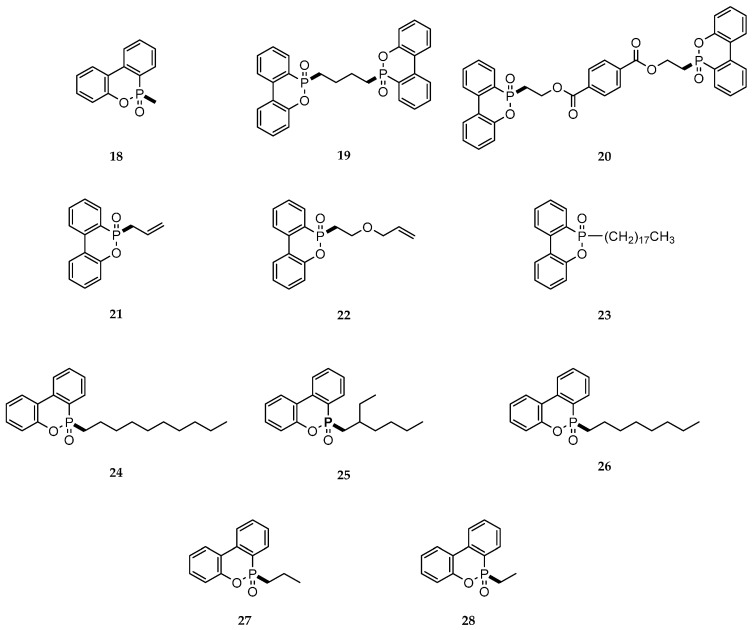
Various DOPO derivatives.

Different DOPO-based derivatives (Figure 11) have been synthesized using the Michaelis–Arbuzov reaction with the aim of studying their flame retardant mechanism in PU foams [69]. For example, methyl-DOPO (**18**) was synthesized in 97% yield and incorporated in polyol to obtain the PU foam. Three different concentrations were tested: 5, 7.5 and 10 phpp (parts per hundred parts polyol). Burning test FMVSS 302 has been performed on the produced foams. With increase in the concentration of **18,** the total heat release (THR) of the foam as measured in cone calorimeter decreased, but the total smoke release (TSR) increased, indicating a strong gas flame inhibiting effect of compound **18**.

Methyl-DOPO (**3.18**) and compounds **19** to **28** (Figure 11) can also be synthesized using Michaelis–Arbuzov rearrangement [70]. For example, a new synthetic route involves using the transesterification of phosphonous acid diesters with the corresponding aliphatic alcohols and subsequent catalyzed Michaelis–Arbuzov rearrangement reaction, which leads to the formation of DOPO derivatives **19** to **28.** These structures were developed as halogen-free flame retardant alternatives for application in various polymers. The melting points were measured for compounds **18** (131 °C), **21** (106 °C), **23** (59–61 °C), **27** (108 °C) and **28** (105 °C). No further analytical studies were performed on these compounds. Such compounds can find potential applications in polymers with processing temperature below 200 °C. The synthesis of DOPO derivatives **18** and **28** were also investigated in other studies [71].

**Scheme 3:**
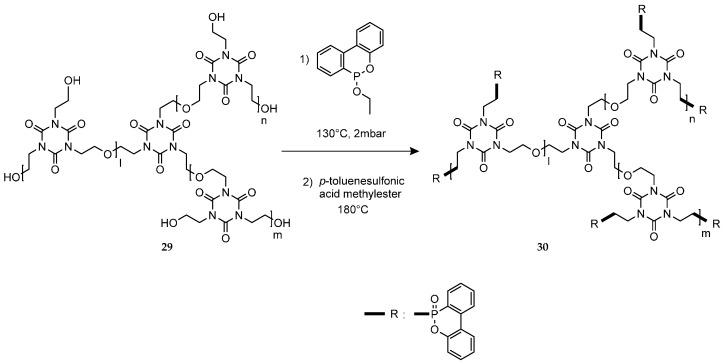
Synthetic route for oligomer **30**.

Compound **30** (Scheme 3) was synthesized via Michaelis–Arbuzov rearrangement reaction with a yield of 82% and applied as flame retardant additive in epoxy resin [72]. TGA and UL 94 test were performed on the resin samples. It was observed in TGA measurements that there was only a slight difference in T_d5%_ between the neat epoxy resin (320 °C) and resin with the compound **30** (340 °C), which indicates a possible gas phase action for this flame retardant. V-0 rating was obtained for epoxy formulation containing compound **30**. It was inferred in this work that the lower the oxidation state of the phosphorus compound, the better is its flame retardancy.

**Scheme 4:**
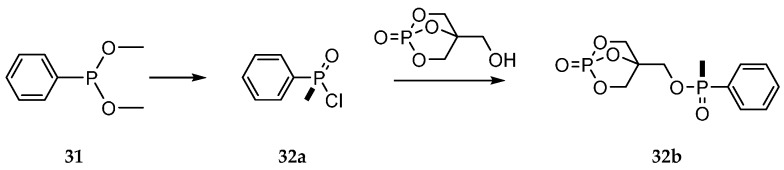
Schematic route for compound **32b** synthesis.

The spiro-cyclic phosphorus-containing flame retardant **32b** was synthesized from reaction of 5-hydroxymethyl-5-methyl-2-phenyl-1,3,2-dioxaphosphorinane-2-oxide with compound **32a** (Scheme 4), in 70% yield [73]. P-C bond in compound **32a** was formed via Michaelis–Arbuzov reaction using the starting material **31** followed by chlorination reaction using thionyl chloride. The final product **32b** was then blended with ABS in 20 wt % and 30 wt % loading and the thermal decomposition and flammability of the formulations were investigated using TGA and UL 94 tests. A 20 wt % loading of **32b** in ABS didn’t show any improvement in flame retardancy. However, for a higher concentration of **32b** (30 wt %), the temperature of 10% weight loss (T_d10%_) was 402 °C and 384 °C under N_2_ and air, respectively. For the neat ABS, a T_d10%_ of 326 °C and 306 °C in N_2_ and air were observed, respectively. For the 30 wt % **32b** formulation, a PHRR of 268.4 W/g and a THR of 26.9 kJ/g (the neat ABS having showed a PHRR of 505 W/g and a THR of 32.7 kJ/g) was observed. It was suggested that this flame retardant acts in a condensed phase when incorporated in PC and in a gas phase when incorporated in ABS.

For a formulation of 30 wt % of compound **32b** in ABS resin, one could achieve a V-0 rating. V-0 classification is the highest classification in terms of fire safety in UL 94 test. However, for the PC blend, a UL 94 V-0 rating was obtained with a much lower concentration (4 wt %) of compound **32b**.

**Figure 12:**
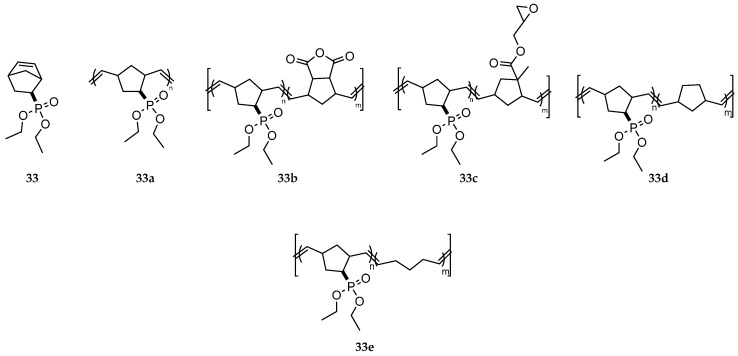
Monomer **33** and its corresponding polymers.

The norbornene-based phosphorus containing compound **33a**–**33e** (Figure 12) were synthesized using ring opening metathesis (ROMP) and investigated as flame retardants [68]. However, the main block monomer **33** has been synthesized in multistep reactions including Michaelis–Arbuzov rearrangement in 68% yield. The polymers **33a**–**33e** were then applied by coating to folded printed paper and subsequent burning tests were performed. The TGA results of these polymers showed that most of them (**33a**, **33b**, **33d**, **33e**) work in condensed phase: the residual weights were 31 wt %, 34 wt %, 27 wt %, 38 wt %, respectively, whereas, for **33c**, a residual weight of 21 wt % was observed. These polymers were also subjected to photolithography using “thiol-ene click” chemistry, which allowed the phosphonated polymer to get a promising negative photoresist. TGA was performed on the polymer samples and homopolymer of **33a** exhibited a two-step thermal decomposition. A T_d10%_ of 331 °C was observed, which was 50 °C lower than for other polymers studied. However, the obtained coating from compound **33a** showed high flame resistance. This was attributed to higher residual weight (37 wt %) for the treated paper as observed in the TGA experiments. LOI value of the blank paper was 21% with a flame speed of 1 cm/s. With 40 wt % of **33a** homopolymer coating on paper, the LOI value was increased to 25% with a burning speed <0.1 cm/s. At a oxygen concentration 35%, the homopolymer **33a** coated paper showed a burning speed <0.5 cm/s, which was half of that obtained for the blank paper. The norbornene derived phosphonated polymer was tested for the first time as flame retardant and the study confirmed its self-extinguishing ability when applied on paper.

**Figure 13:**
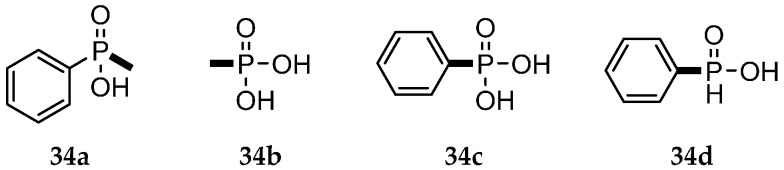
Flame retardant **34a**–**34d** structures.

Methylphenylphosphinic acid (**34a**, Figure 13) was obtained from hydrolysis of methyl methylphenylphosphinate (MMPP) in hydrochloric acid in 90% yield. MMPP was synthesized in a two-step synthetic strategy. It involved reactions of dichlorophenylphosphine with methanol to form dimethyl phenylphosphonite and subsequent rearrangement reaction with methyl iodide to yield MMPP [74], in 65% overall yield. It was observed that the thermal decomposition of compound **34a** occurred in one step compared to other structurally analogues flame retardants studied (**34b**, **34c** and **34d**, Figure 13). In addition, the compound **34a** showed a T_d10%_ < 300 °C in the TGA experiment. The flame retardant additives were also copolymerized with acrylonitrile-butadiene-styrene (ABS) and ethylene-vinyl acetate (EVA) separately with a 20 wt % loading (~4 wt % phosphorus). Based on the residue obtained in TGA, it was reported that all these flame retardants act in the gas phase in EVA formulation, compounds **34a** and **34b** act in gas phase in ABS formulations and compounds **34c** and **34d** act in both gas and condensed phases in ABS formulations. A UL 94 V-0 rating for formulation containing 20 wt % of compound **3.34a** in ABS was observed. Compounds **34a**, **34b**, **34c** and **34d** were compared for their flame retardant performance. It was observed that compound **34b** exhibited the best flame resistance; however, it was considered not suitable as real flame retardant because of its high solubility in water. Compound **34a** exhibited the second best result in the UL 94 test, was also the most volatile, and followed by **34c** and **34d**. A general conclusion on the influence of the structure on the flame retardancy was made i.e., flame retardant having a methyl group attached directly to the phosphorus atom showed greater flame retardancy compared to compounds having a phenyl, hydrogen or a hydroxyl group.

### 3.4. Grignard Type Reaction

**Figure 14:**
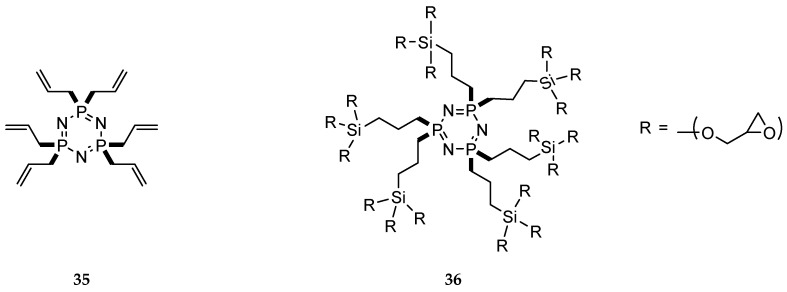
Chemical structures of **35** and **36**.

Grignard reactions can also be used for the creation of new P-C bonds [75]. Hexaallyltriphosphazene (HAPP) (**35**, Figure 14) was prepared from the reaction of allylmagnesium bromide with hexachlorocyclotriphosphazene (in 94% yield) for application in epoxy system to improve its flame retardancy. HAPP was further reacted in two steps to give hexa(3-triglycidyloxysilylpropyl)triphosphazene (**36**) in 92% yield, which was then used in epoxy formulations. LOI tests were performed for the epoxy system with and without flame retardant additive **36**. The results showed that compound **36** played a major role in improving flame retardancy. For example, the LOI value of the neat resin was 22.5%, whereas, with 15 wt % of **36**, the LOI value increased to 32.1%. Moreover, the TGA data revealed that the higher the concentration of compound **36** in the epoxy system, the higher the T_d5%_ and T_d50%_ temperatures. The TGA results also showed that, for the most concentrated resin (15 wt % of compound **36**), the char residue under was 10.2%. Therefore, it was reported that this flame retardant acts predominantly in the gas phase.

**Figure 15:**
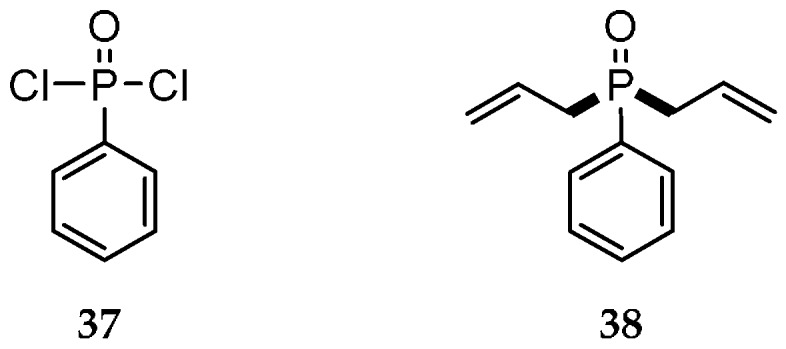
Phosphine oxide derivative **38** and its precursor **37**.

Diallylphenylphosphine oxide (**38**, Figure 15) was prepared from reaction of allylmagnesium bromide with phenylphosphonic dichloride (**37**, Figure 15), in about 55% yield [76]. The light yellow product obtained was applied in UV-cured organic–inorganic hybrid coatings on corona treated Plexiglass^®^ panels. The formulations contained tetraethyl orthosilicate (TEOS), diallylphenylphosphine oxide (5 to 20 wt %), an aliphatic urethane diacrylate resin and a silane-coupling agent. Free films on Teflon™ wells were also prepared and cured with UV-light. Mechanical tests were performed on the cured samples with and without addition of compound **38** and evaluated for tensile properties like the Young’s modulus and the elongation at break, the contact angle, the pendulum hardness and the cross-cut adhesion. It was found that the addition of diallylphenylphosphine oxide increased the Young’s modulus but decreased both the tensile strength and the elongation at break compared to the formulation without the compound **38**. The contact angle also decreased with the addition of compound **38** to the formulation due to its high polarity. The pendulum hardness increased with addition of the flame retardant due to a higher cross-linking density and the cross-cut adhesion (which ranged from 0 for the best and 5 the poorest adhesion) showed a result of 0 for a 15 wt % and 20 wt % flame retardant content. The thermal stability of the samples was also investigated by TGA and it was found that addition of 20 wt % of compound **38** lowered the T_d5%_ by 40 °C compared to the sample without the flame retardant. The char content as measured in TGA for the most concentrated resin (20 wt % of **38**) is lesser for lower (10% of **38**) concentration formulation. Normally for a flame retardant working in the condensed phase, the char content in TGA analysis increases with increase in its concentration in the polymer. Thus, one can infer from the TGA data that flame retardant **38** work mostly in the gas phase. The photo-DSC test was also performed, and it was concluded that the enthalpy of polymerization increased with the increase in concentration of compound **38**.

## 4. Flame Retardants with P-C_aromatic_ Bonds 

In the following sections, we have summarized specific examples of P-C_aromatic_ bond containing organophosphorus compounds, which are classified based on the synthetic strategy for creation of P-C bond. For clarity, the specific P-C bonds that were created in the structure have been marked in bold.

### 4.1. Friedel–Crafts Type Reactions

**Figure 16:**
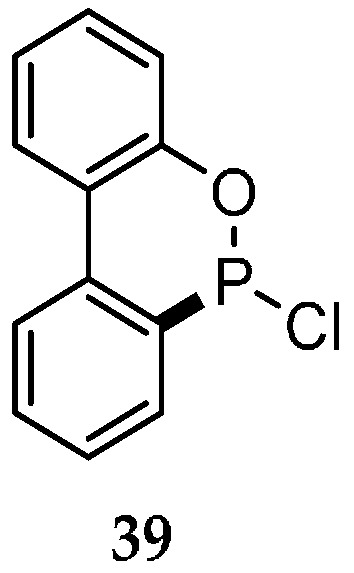
Structure of DOP-Cl **39**.

One of the main routes to phosphorus-aromatic carbon bonds formation is a modified Friedel–Crafts reaction usually involving phosphorus trichloride and a Lewis acid. DOP-Cl (Compound **39**, Figure 16) has reportedly been synthesized starting from *o*-phenylphenol using phosphorus trichloride in presence of zinc chloride catalyst in 95% yield [66,77]. DOP-Cl is an important precursor for DOPO derivatives due to its high chemical reactivity towards nucleophiles [5]. As an example, it is shown in the literature that DOP-OMe reacts with hydroxymethyl substituted phenols via a Michaelis–Arbuzov type rearrangement to give compound **14** (Scheme 2) in 32% yield. Compound **14** has a glass transition temperature of 108.5 °C. In the published patent, it was claimed that this family of compounds can be used as flame retardant for a wide range of polymers such as PU, epoxy and other thermosetting resins or thermoplastics in general. It is further claimed that these compounds are particularly adapted to protective coating formulations, electrical laminates and molded thermoplastic products [77].

**Figure 17:**
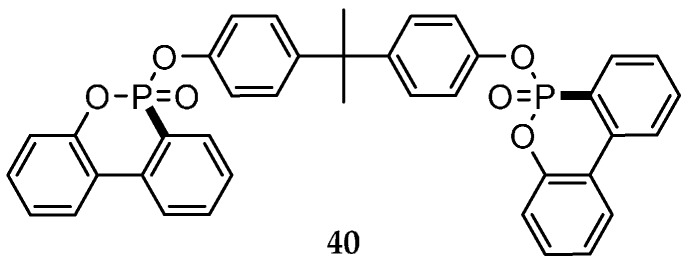
Chemical structure of bisphosphonate **40**.

The same reagents as described earlier were also used in another patent to prepare compound **39** (Figure 16) and was subsequently oxidized [78]. Next, esterification with phenols or polyhydroxybenzenes was performed to develop several flame retardants and one such example is compound **40** (Figure 17) (obtained from bisphenol A). No further information on characterization, flame retardancy or applications were provided.

**Scheme 5:**
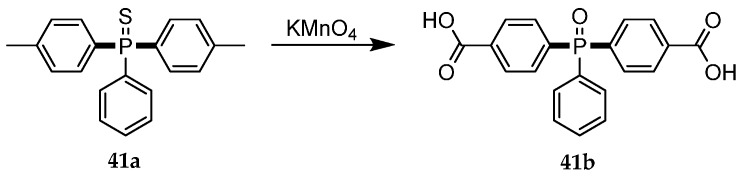
Oxidation of **41a** to **41b**.

Friedel–Crafts reactions can also be performed using aluminum chloride as Lewis acid. It was reported that dichlorophenylphosphine sulfide reacts with two equivalents of toluene in the presence of AlCl_3_ catalyst to give **41a** (Scheme 5, 84% yield) [79]. The compound was further oxidized to give BCPPO (**41b**, Scheme 5). Several polycondensations of this flame-retardant monomer and terephthalic acid (TPA) with bisphenol A were performed using different TPA/**41b** ratios, yielding six different polyarylates. Films were prepared from these phosphorus-containing polyarylates for further characterization. Mechanical properties of the polymers were evaluated, and it was found that tensile strength and elongation at break decreased with introduction of **41b** in the polyarylate. Crystallinity was lost when **41b** was incorporated in the polymer. Glass transition temperature, T_d5%_ temperature and char yield followed the same positive trend when phosphorus content was increased, showing tangible evidence that **41b** introduction can improve the thermal stability of the resulting polymer. To confirm improved flame retardancy of the new materials, LOI value was measured and was shown to increase from 30.7% to 34.5% with increasing phosphorus content. Further study of the thermal decomposition behavior of the polymers concluded that increasing the proportion of compound **41b** in the polymer increased the char formation, indicating that compound **41b** acts in both the gas and condensed phase. Finally, an additional goal of this work was to study the solubility of the synthesized polyarylenes. Good solubility was achieved in a wide selection of organic solvents for formulations with 20% or more of compound **41b**. This was attributed to the increase of free volume induced by the introduction of bulky pendant phenyl units in the chain. It was thus concluded in this work that introduction of compound **41b** enhanced the thermal stability while improving the polyarylates’ processability.

**Figure 18:**
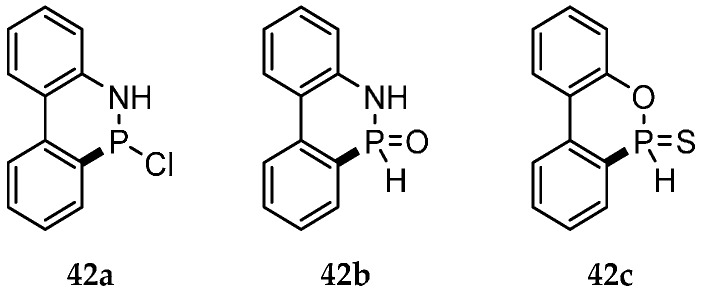
DOPO analogues.

DAP-Cl (compound **42a**, Figure 18), a precursor to the nitrogen-containing DAPO (compound **42b**, Figure 18), was synthesized in 95% yield using 2-aminobiphenyl, PCl_3_ and AlCl_3_ as catalyst [80,81]. It was reported that DAPO exists in the solution in a tautomeric equilibrium with its corresponding phosphonamidic acid [82]. Another DOPO derivative, DOPS ( compound **42c**, Figure 18), was synthesized by reacting DOP-Cl with hydrogen sulfide at room temperature (89% yield) [83]. Epoxy phenol novolac resins were functionalized with DAPO and DOPS, followed by a curing step. Phosphorus content in these formulations ranged from 0.6 to 1.6 wt %. DSC measurements showed that DAPO-modified resins had higher T_g_ compared to the reference DOPO-modified resins. This was attributed to the presence of an amine group that can also act as a cross-linking reagent, resulting in a more rigid network. On the contrary, DOPS-modification induced a sharp decrease of T_g_, which can result in lower crosslinking density due to the bulky sulfur atom. Flame retardancy of these modified resins was investigated and compared to DOPO-modified resins. At 1.6 wt % P loading, DOPS- and DAPO-modified resins achieved UL 94 V0 rating. Char yield in TGA experiments reached 35% for DAPO and 32% for DOPS based resins. Nitrogen-phosphorus synergistic effect was considered to be the reason behind increased char yield, and was also presented as a possible explanation for their slower decomposition rate. Sulfur-phosphorus synergy hasn’t yet been extensively studied so no conclusion could be drawn. Increased char yield indicate a high probability that DOPS and DAPO may work in condensed and gas phase, whereas DOPO acts mainly in gas phase.

**Scheme 6:**
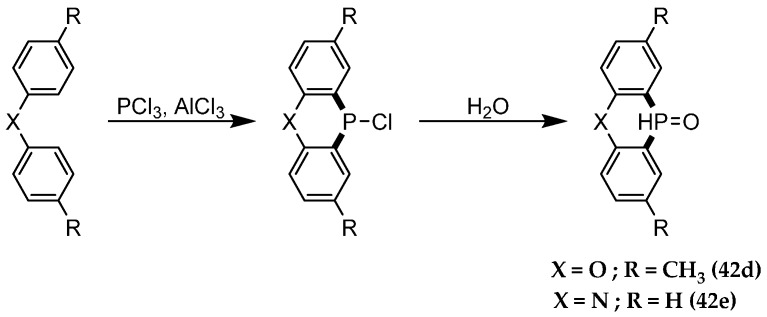
Synthetic route for phosphine oxides DPPO (**42d**) and DPPA (**42e**).

Two similar compounds were prepared using aluminum chloride and phosphorus trichloride reagents and di-*p*-tolylether [84] or diphenylamine [85], affording DPPO (compound **42d**, 91% yield) and DPPA (compound **42e**, 61% yield), respectively (Scheme 6). Compound **42d** was then oxidized with hydrogen peroxide or reacted with formaldehyde, affording DPPO-OH and DPPO-CH_2_OH, respectively. These two intermediates along with unmodified **42d** were then used to functionalize epoxy novolac resin DEN 438 by their reaction with the epoxy groups, resulting in DPPO-OH-DEN, DPPO-CH_2_OH-DEN and DPPO-DEN. The same procedure was applied to DOPO, affording DOPO-OH, DOPO-CH_2_OH-DEN and DOPO-DEN. Each flame-retardant prepolymer was prepared with phosphorus content ranging between 0.5% and 2.0%. 4,4′-Diaminodiphenylmethane (DDM) was used as hardener during the curing step.

Compound **42e** was directly reacted to epoxy groups of DGEBA resin (via the phosphorus and nitrogen atoms) to obtain DPPA-DEN prepolymers [85]. The phosphorus content of the formulations varied from 0 to 3.20 wt %. Curing was then performed using DDM or 3,5-diethyltoluene-2,6-diamine (DETDA) as hardeners. High level flame retardant properties were achieved for all the DOPO- and DPPO-based epoxy resins. At a 0.81 wt % phosphorus content, all of them acehieved UL 94 V-0 rating. For this same phosphorus content, DOPO-based resins exhibited LOI value between 31% and 31.5%, whereas, for DPPO-based resins, LOI values ranged between 27.4% and 28.3%, both considered really good values for flame retardants. In case of compound **42d**, PO radicals were shown to form in the gaseous phase, indicating the gas phase mode of action [84]. DPPA-DEN resins exhibited lower flame retardant performance, needing a phosphorus content of 3.20% to attain UL 94 V-0 rating as well achieving LOI values 28.5% (DDM-cured) and 29.3% (DETDA-cured). Thus, it was concluded that DPPO-, DPPA- and DOPO-functionalization proved to be beneficial in improving flame retardants in epoxy resins.

**Figure 19:**
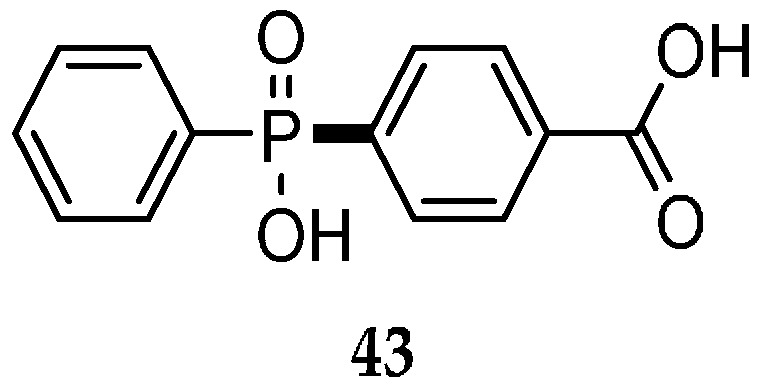
Chemical structure of compound **43**.

4-carboxyphenylphenylphosphinic acid (**43**, Figure 19) was prepared by addition of toluene to dichlorophenylphosphine and anhydrous aluminum chloride followed by hydrolysis and the oxidation reaction [86], achieving a yield of 33.1%. Its solubility in water was investigated from 26.70 °C to 94.52 °C and experimental data was correlated using a second-order polynomial with an absolute average deviation of 2.1%. These solubility measurements were considered of interest because of the important role of water in the industrial production and purification of compound **43**. Being a reactive phosphorus-containing monomer, compound **43** can be used as a co-monomer for manufacturing functional polyesters.

### 4.2. General Substitution Reactions

**Figure 20:**
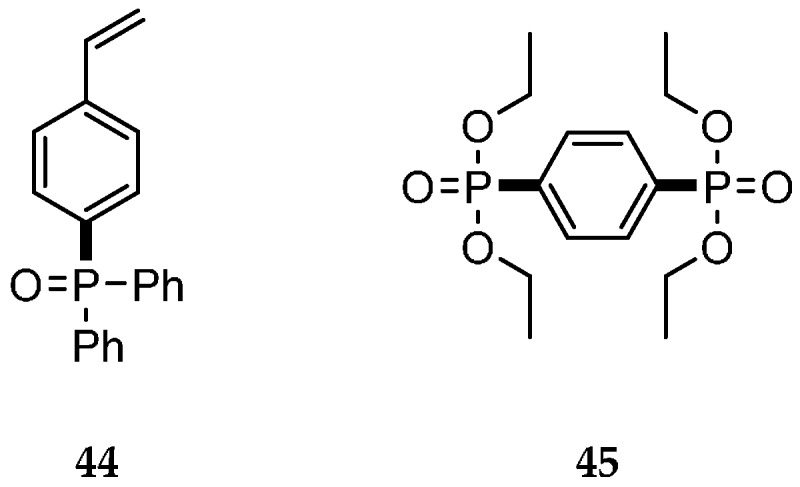
Phosphine oxide derivative **44** and phosphonate derivative **45**.

Compound **44** (Figure 20) was reported to be synthesized by adding chlorodiphenylphosphine to a mixture of *p*-bromostyrene and butyllithium, followed by an oxidation step, resulting in 65% yield [87]. Cationic copolymerization was investigated with soybean oil for different ratios of styrene to substance **44**, using divinylbenzene as crosslinker. Due to a problem of heterogeneity before and after the polymerization reaction, an alternative phosphorus-containing styrene substance was investigated. This alternative compound underwent cross-metathesis with a vegetable-oil derivative and was copolymerized, affording homogeneous thermoset polymers. Thermal-oxidative degradation was investigated by TGA, showing that 1 wt % phosphorus incorporation improved the thermal stability of the thermoset slightly. Condensed phase action was inferred from the increased char formation observed in the TGA. Phosphorus-free reference polymer achieved 19.2% LOI value, and phosphorus-containing polymer was reported to achieve a maximum LOI value of 24%.

1,4-bis(diethylphosphoro)benzene (**45**, Figure 20) was synthesized from the reaction of 1,4-dibromobenzene with triethyl phosphite [88]. Compound **45** was used in battery electrolytes compositions to enhance flame retardancy properties and to reduce impact of possible thermal runaway.

Phosphorus-carbon bonds formation were reported with good yields for substitution reactions involving triethyl phosphite and cyanuric chloride. These compounds are considered very efficient as charring agents due the presence of nitrogen in the triazine ring and thus expected to exhibit P-N synergism.

**Figure 21:**
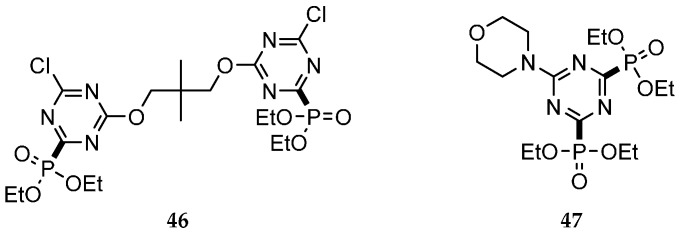
Triazine derivatives **46** and **47**.

In a recent work [89], diethyl phosphite-functionalized cyanuric chloride (86.1% yield) was dimerized using neopentyl glycol as a bridging agent, yielding compound **46** (Figure 21, 92.5% yield). The target flame retardant can bind covalently to cotton fabrics by reaction of the cellulose hydroxyl groups via a substitution reaction. The best curing method was established from several trials and was applied to treat cotton fabric samples with 10 to 30 wt % of the flame retardant. TGA analysis showed an improvement of thermal stability and increase of the char yield above 30% for all treated samples, indicating the condensed phase mode of action of the flame retardant. During vertical flammability testing, char length became shorter with increasing flame retardant content, and a B-1 rating was achieved for samples treated with more than 20 wt % flame retardant. Improved flame retardancy was confirmed from LOI tests, which showed a LOI value of 30.3% and 18% for 30 wt % of flame retardant treated fabric and untreated fabric, respectively. Washing with water of treated cotton showed a rapid decrease in LOI values. Initial LOI of 28.4% dropped to 22% after 10 times washing indicating not all flame retardants reacted with the cellulose. Such flame retardants primarily act in the condensed phase in cellulose based materials.

A reaction of three equivalents of triethyl phosphite with cyanuric chloride resulted in 2,4,6-trisubstituted 1,3,5-triazine (86.1% yield). One of the diethyl phosphate groups on the triazine ring was then substituted by morpholine, producing another substituted triazine-based compound **47** (Figure 21, 92.5% yield) [90]. An oligomer was synthesized by transesterification reaction of compound **47** with pentaerythritol in 93.6% yield. It was then incorporated in polypropylene at 10 and 30 wt %, which respectively achieved UL 94 V-2 and V-0 ratings, while LOI values of 23.4% and 28.9% were observed for the same formulations, respectively. A polymer formulation was further prepared with 30 wt % ammonium polyphosphate/pentaerythritol/**47** (ratio 3:1:2), which achieved a higher LOI of 29.4% and UL 94 V-0 rating. This improvement in flame retardancy was attributed to the possible synergistic effect between the three additives.

### 4.3. Addition Reactions

**Scheme 7:**
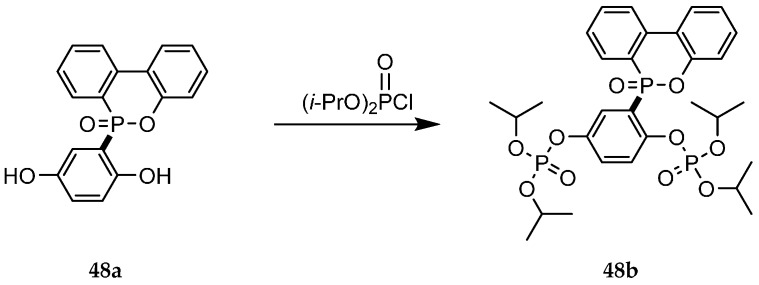
Synthesis of DOPO based phosphinate derivative **48b** from **48a**.

Recently in the literature, a large amount of research work can be found on addition reactions of DOPO derivatives on α,β-unsaturated ketones. This is mainly due to the versatility and high yields allowed by this type of reaction. DOPO addition on 1,4-benzoquinone is one of the most common uses of this addition reaction. DOPO-BQ (compound **48a**, Scheme 7) was synthesized following a previously reported method in 93% yield [91] as an intermediate leading to six flame retardant additives for lithium ion batteries. They were then characterized for their thermal properties using TGA and DSC [92]. From these six compounds, the compound **48b** (Scheme 7) that achieved a T_d5%_ of 210 °C was selected for further flame retardant evaluation. It was mixed with conventional Li-ion electrolytes, with weight fractions ranging from 2.5 to 25 wt %. Self-extinguishing time (SET) measurements were performed, showing a sharp decrease in SET, whereas ionic conductivity underwent a 50% decrease when increasing the weight fraction of the additive from 2.5 wt % to 25 wt %. The flame retardant efficiency of the electrolyte formulations was confirmed, but lower battery performances are to be expected due to lower ionic conductivity results. Char formation indicated a condensed phase action. Studies on actual battery performances along with electrochemical properties are underway.

**Figure 22:**
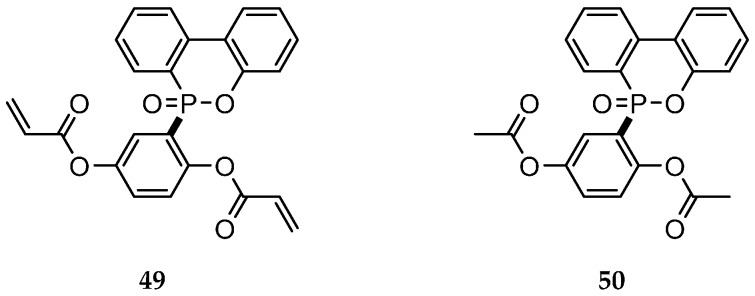
DOPO based phosphinate derivatives **49** and **50**.

Compound **48a** was reacted with acryloyl chloride to obtain a phosphorus-containing acrylate monomer (compound **49**, Figure 22) [93]. Various concentrations (0 to 20 wt %) of this compound were introduced into the formulation of unsaturated polyester resins synthesized by radical copolymerization. A high LOI value of 24% was achieved for formulations containing ≥5 wt % phosphorus-containing monomer. T_d5%_ peaked at 260 °C in air, at 300 °C in nitrogen, for the highest flame retardant-containing formulation, and its peak heat release rate (PHRR) was reduced to 288.7 W/g. Its flame retardant properties were confirmed both in condensed and gas phase, due to charring and flame inhibition phenomena, respectively.

Substance **48a** was also reacted with acetic anhydride, affording **50** (Figure 22, 75% yield) [94]. Substance **50** was then copolymerized with *p*-acetoxybenzoic acid, terephthalic acid and the condensation product of terephthalic acid with 1,8-octanediamine, producing a new flame retarded thermotropic liquid crystal polymer. DSC experiment showed lower T_g_ and T_m_ for it compared to usual liquid crystal polymers, namely, 127 °C and 147 °C. It adopted nematic liquid crystalline behavior in the temperature range 180–330 °C. Thermal stability of the polymers was investigated by TGA and T_d5%_ was estimated at 385 °C, showing satisfying thermal stability and char yield, allowing for very good flame resistance.

**Figure 23:**
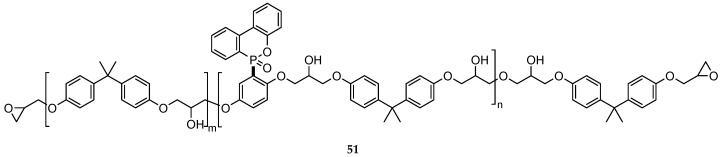
DOPO-BQ (**48a**) functionalized DGEBA resin **51**.

Diglycidyl ether of bisphenol-A epoxy resin (DGEBA) was modified by compound **48a**, affording the phosphorus-containing epoxy resin **51** (Figure 23) [95]. Four cured epoxy resin/polyhedral oligomeric silsesquioxane (POSS) hybrids were synthesized, and all samples contained 2 wt % phosphorus but with varying silicon content (from 0 to 4 wt %). The thermal-oxidative stability of the polymers was evaluated by TGA. With increasing the silicon content, TGA analysis showed a decrease of T_d5%_ and an increase of T_d50%_ and char yield, which was attributed to the phosphorus-silicon synergism. Further flame retardant properties were estimated by PCFC, giving evidence that PHRR decreased and occurred at higher temperatures with increasing silicon content. T_g_ of the polymer was shown to increase with increase in phosphorus and silicon content. SEM and XPS were used to analyze char residues and results obtained reinforce hypotheses on phosphorus-silicon synergistic impact on epoxy resins flame retardancy.

Similar to the previous study, different epoxy resin **51** were synthesized from reaction of DGEBA and compound **48a** in different molar ratios [96] and several dicyanate esters were used as curing agents. TGA data under nitrogen showed evidence that degradation temperature and char yield for the resin increased with the increasing phosphorus content, showing a condensed phase mode of action. UL 94 V-0 rating was attained for formulations containing around 2 wt % phosphorus, when UL 94 V-1 rating was achieved for the resins containing around 1 wt % phosphorus, the curing dicyanate ester having no major influence. Potential applications as flame retardant laminates or protective coatings were suggested for these resins.

**Figure 24:**
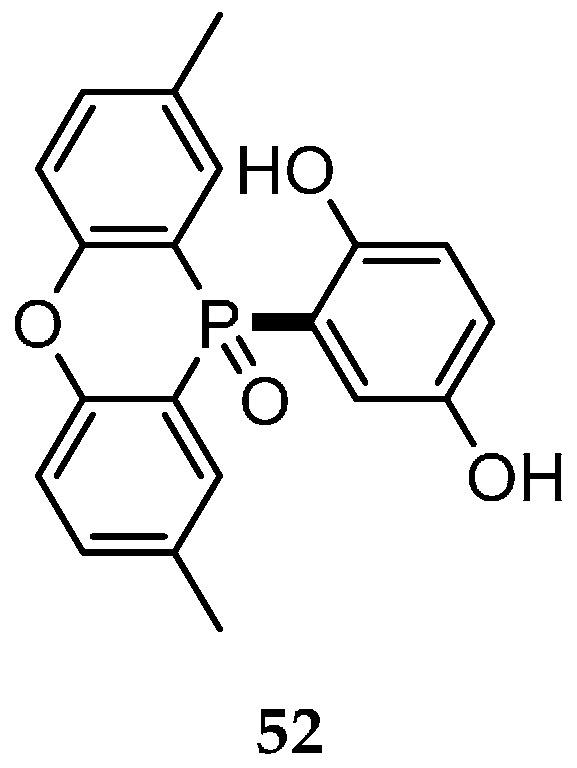
Phosphine oxide derivative **52**.

Previously synthesized compound **48a** [91] and DPPO-HQ, (compound **52**, Figure 24) (obtained by reacting compound **42d** (Scheme 6) with *p*-benzoquinone) [84,97] were used as diols to form aromatic polysulfones (PSU) by nucleophilic aromatic substitution polycondensation with 4,4′-difluorodiphenylsulfone (DFDPS) [98]. Two polysulfones were thus obtained, having around 5 wt % phosphorus content. Both exhibited significantly higher T_g_ than phosphorus-free, bisphenol A-based PSU (reference), and a two-step decomposition was observed instead of one-step for the reference polymer in TGA. TGA and Py/GC-MS data were used to formulate a hypothesis concerning the thermal degradation process of these formulations. It was also concluded that DOPO-based PSU combined gaseous and solid-state flame-retarding mechanisms, whereas DPPO-based PSU favored solid-state action. Thus, DOPO-based PSU was considered as a slightly better in terms of flame retardancy.

**Scheme 8:**
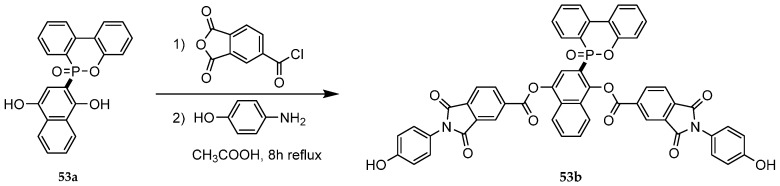
Synthetic route for DOPO based phosphinate **53b**.

Following the same reaction principle as described earlier, DOPO can also react with 1,4-naphthoquinone to give DOPO-NQ (compound **53a**, Scheme 8, 70% yield) [99], which was then functionalized by trimellitic anhydride chloride and 4-aminophenol to give a bisphenol derivative (compound **53b**, Scheme 8, 71.8% yield) [100]. Several liquid crystalline co-poly(ester imide)s were prepared by polycondensation of an aromatic diacid chloride with various ratios of the compound **53b** and aliphatic diols. Thermal analysis of the polymers was studied by TGA, and it was observed that all polymers followed a two-step decomposition process. The least stable polymer exhibited a T_d5%_ of 340 °C, whereas the most stable showed a T_d5%_ of 395 °C. The thermal degradation behavior of the polymers was investigated by FTIR-TGA and SEM-TGA analysis. Phosphorus-nitrogen synergy is expected to contribute to flame retardancy, suggesting gas and condensed phase activity for the phosphorus compounds. Solubility in various solvents was investigated. Liquid crystalline properties were confirmed by XRD and DSC analysis.

In a second paper [101], the same group synthesized three different poly(ester imide)s by solution polycondensation of equimolar compound **53b** (Scheme 8) and aromatic diacids (either terephthalic acid, isophthalic acid or 4,4′-dicarboxydiphenylether) yielding PEI-1, PEI-2 and PEI-3, respectively. TGA experiments were run and concluded that these polymers exhibited 55% char and were stable up to 375 °C. Their T_g_ were respectively measured at 226 °C, 200 °C and 190 °C, making them good candidates for thermoforming. Energy-dispersive X-ray spectroscopy (EDX) confirmed the presence of phosphorus in the char, proving condensed phase action. Liquid crystalline properties were confirmed by WAXD, optical properties and DSC studies.

**Figure 25:**
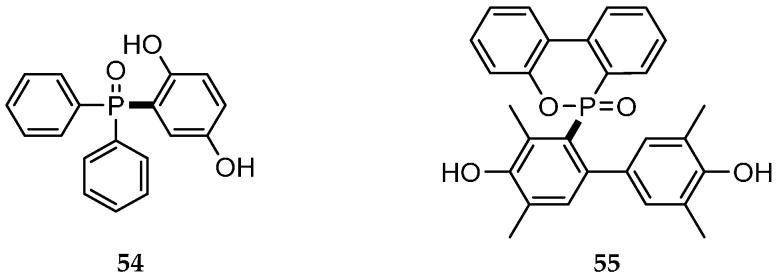
Chemical structures of compounds **54** and **55**.

Diphenylphosphine oxide was reported to react with benzoquinone to give compound **54** (DPO-BQ, Figure 25, 78% yield) [102]. Its thermal stability was shown to be slightly better than the reference compound **48a**, having a T_d5%_ of 315 °C and a slower decomposition in air. The compound **54** was then reacted to epoxy resin, followed by curing with 4,4′-diaminodiphenylsulfone to achieve thermoset resins having 1 and 2 wt % phosphorus. TGA investigations showed few differences between the two formulations containing various concentrations of compound **54**, T_d5%_, being around 391 °C in nitrogen and 384 °C in air. Polymer formulation with 2 wt % phosphorus achieved a UL 94 V-0 rating and LOI of 32.4%, whereas the one containing only 1 wt % phosphorus reached a V-1 rating and a LOI value of 29.2%. Good flame retardance was achieved at low phosphorus loadings, although TGA results showed little improvements compared to phosphorus-free epoxy resins, except for higher char yields, indicating a condensed phase mode of action.

A recent patent reported that DOPO addition on 3,3′,5,5′-tetramethyldiphenoquinone gave a yellow compound **55** (Figure 25) in 73% yield [103]. The aim of this work was to synthesize phosphorus-containing compounds with low steric hindrance for application in flame retardant epoxy resins.

**Figure 26:**
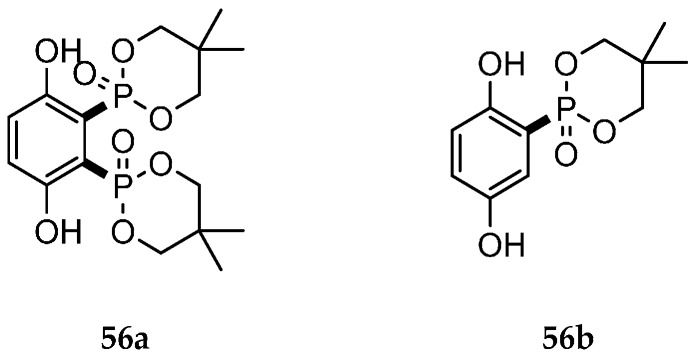
Phosphonate derivatives **56a** and **56b**.

According to previous studies, a disubstituted phosphorus compound **56a** and a monosubstituted phosphorus compound **56b** (Figure 26) were obtained as a mixture using modified separation and purification steps. Melting points for these compounds were 258–260 °C and 184–187 °C, respectively [104]. Further characterizations were performed and were compared with other phosphorus compounds described later in the text.

### 4.4. Michaelis–Arbuzov Rearrangement

**Figure 27:**
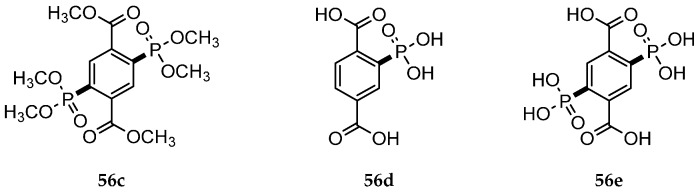
Chemical structures of compounds **56c** to **56e**.

In the same work [104], phosphonoterephthalic acid derivative **56c** (Figure 27) was synthesized using a Michaelis–Arbuzov rearrangement from a previously published synthesis protocol [105]. Compounds **56d** and **56e** (Figure 27) syntheses were also based on a previous study from the same group (exhibiting m.p. of 296–298 °C and 310 °C, respectively). The syntheses involved iodoester reagents with palladium catalyst in a yield of around 80%. PU foams were prepared with compounds **56a** to **56e** according to two different processing methods: *Preps* (reactive flame retardant copolymerized during PU foam formation) and *Blends* (10 wt % of flame retardant additive used with already prepared PU). These foams were analyzed for thermal and flame retardant behavior using TGA and PCFC, respectively. It was found that compound **56e** did decrease the heat release and inhibited melting and flowing of the PU in the *Blend* form. In the *Prep* form, the addition of this compound did not affect the heat release. The resulting PU formulation also had a two-step thermal degradation and increased char yield at 800 °C (neat PU around 9% and PU with 10 wt % flame retardant around 29%). For compound **56d**, the *Prep* form didn’t affect either the heat release, but, in the *Blend* form, it induced a significant decrease of the THR. One possible explanation of this phenomenon was the incomplete incorporation of the flame retardant in the PU foam. Compound **56c** showed the same flame retardant behavior as **56e** for PU prepared by *Blend* as well as in *Prep* forms. The previously mentioned phosphonoterephthalic acid derivatives **56a** and **56b** have shown similar efficiency in decreasing the total heat release in both processing methods. It was also found that they didn’t inhibit melting and flowing of the PU. In conclusion, compounds **56c** and **56e** showed the best flame retardancy properties of all the investigated structures.

### 4.5. [1,3]-Sigmatropic Rearrangement

**Figure 28:**
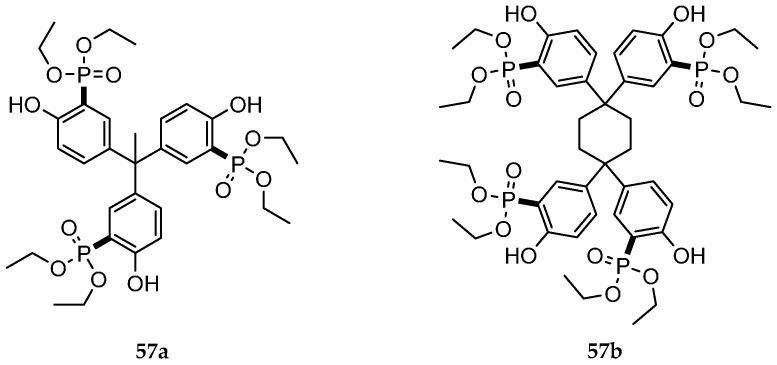
Phosphonate derivatives of polyphenols **57a** and **57b**.

Two polyphenols were transformed into corresponding phosphates using diethyl phosphite following Atherton–Todd reaction [106]. Further treatment with lithium diisopropylamine allowed breakage of a P-O bond and migration of the phosphorus to create a new P-C bond. This process known as [1,3]-sigmatropic rearrangement yielded two different compounds, **57a** (62% yield, m.p. 169–171 °C) and **57b** (65% yield, m.p. 216–217 °C), and both were obtained as white crystals. Their structures are shown in Figure 28. Although no flame retardant tests were performed, it was concluded that these phosphorus-containing polyphenols could be introduced as flame retardant monomers in high-performance polymers including PEEK, PEES, PEK, PES or PC.

**Scheme 9:**
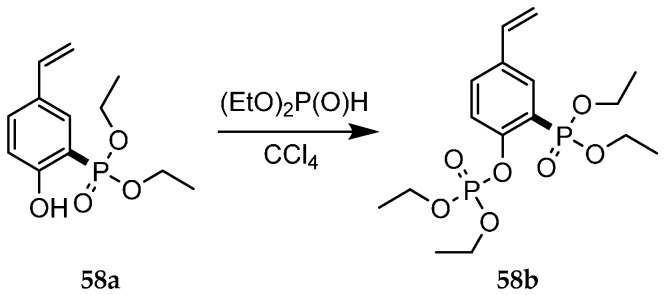
Synthesis of the phosphorus-containing vinyl monomer **58b** from **58a**.

According to the same mechanism, the phosphonate product (diethyl-[2-hydroxy-5-vinylphenyl) phosphonate (compound **58a**, Scheme 9) was synthesized starting from diethyl-(4-vinylphenyl)phosphate and obtained in a yield of 82% [107]. Diethyl phosphite was then used to react with the hydroxyl group using Atherton–Todd reaction, yielding compound **58b** (Scheme 9, 85.7% yield). Compound **58b** was copolymerized with styrene using three different compositions (5, 10 and 20 wt % of monomer **58b**) yielding copolymers that T_g_ decreases from 101 to 95 °C when flame-retardant monomer mass fraction is increased in the polymer formulation. TGA showed a two-step degradation behavior explained by the presence of diethyl phosphate and diethyl phosphonate, allowing the main degradation step to happen at a temperature at least 60 °C higher than the neat polystyrene. Copolymer incorporation lowered the temperature of the first decomposition step but increased the temperature of the second degradation stage. The char yield increased from 1.1% to 7.3% with increasing the phosphorus-containing monomer, showing evidence of a condensed phase mechanism. PCFC measurements followed a similar trend, the total heat release decreasing from 40.1 to 38.2 kJ/g with increasing phosphorus content. As a conclusion, efficient flame retardant vinyl polymers were obtained by copolymerizing phosphorus-functionalized styrene with styrene units.

### 4.6. Phospho-Fries Rearrangement

**Figure 29:**
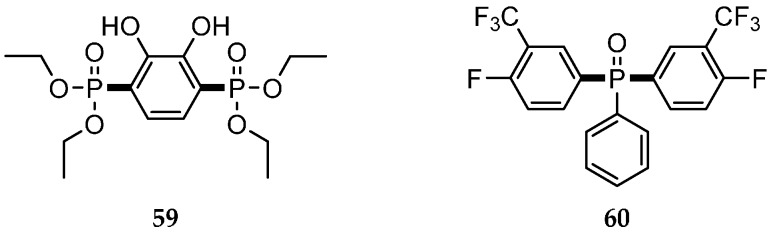
Chemical structure of compounds **59** and **60**.

A phosphorus version of the Fries rearrangement was reported by treating tetraethyl-1,2-phenylene bis(phosphate) with a mixture of butyllithium and lithium diisopropylamide to produce diphosphonate compounds **59** (Figure 29, 67% yield) [108]. This procedure was a modified version of a synthesis reported in a previous publication [109] explaining that phosphate esters in presence of an organolithium compound form anions that undergo phosphorus migration from oxygen to carbon. Compound **59** was an intermediate to four lithium salts: three boron-centered anions and one phosphorus-centered. Pyrolysis combustion flow calorimetry (PCFC) results showed evidence that lithium boron phosphorus complexes reached high char yield (up to 49.60%), which lowers the peak heat release rate temperature below 312 °C. These high char yield values indicate condensed phase activity, and could even indicate phosphorus-boron synergism. TGA analysis completed this study by proving these new lithium salts were stable up to 200 °C. It was concluded these new compounds should be effective flame retardants for lithium ion batteries thanks to their reduced flammability, either as electrolyte additives or as possible alternative to existing lithium salts.

### 4.7. Grignard Type Reactions

Grignard reagents represent an additional method of P-C bonds synthesis in which the nucleophilic carbon can react as a base. 5-bromo-2-fluorobenzotrifluoride was added to magnesium turnings, forming the associated organomagnesium compound. Phenylphosphonic dichloride was then added to yield compound **60** (Figure 29) as a pale yellow solid (80% yield) [110]. Polymerization reactions were carried out using an equimolar ratio of compound **60** and different diphenol derivatives. Six novel poly(arylene ether phosphine oxide)s were obtained, with T_g_ in the range of 173–252 °C, and T_d5%_ values being between 457 °C and 490 °C. PCFC investigation showed great influence of the diphenol structure, and the peak heat release rate varied from 184 to 67 W/g. Films were prepared, resulting in transparent, brittle or flexible films depending on the diphenol monomer used. The mechanical properties of the flexible films were evaluated, tensile stress reaching up to 54 MPa, Young’s modulus up to 0.97 GPa and elongation at break up to 47%. This study brought to light compounds with interesting thermal stabilities, and choosing the right diphenol monomer can allow to finely tune the poly(arylene ether phosphine oxide) properties.

**Scheme 10:**
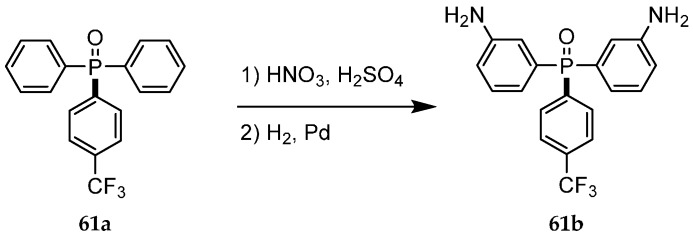
Two-step amination of **61a** to yield **61b**.

In a similar approach, diphenylphosphinic chloride was reacted with 4-(trifluoromethyl)phenylmagnesium bromide to yield compound **61a** (Scheme 10) [111] having a melting point of around 90.5–91.2 °C. This product underwent nitration followed by hydrogenation to give the amino derivative **61b** (Scheme 10). This phosphorus-containing diamino compound can be used to prepare polyimides, polyamides that exhibit very good fire retardancy, thermal stability, mechanical properties, adhesion as well as low dielectric constant. Possible applications would be adhesives for metals, semiconductor packaging or optical materials.

## 5. Conclusions

P-C bond creation and its use in synthetic chemistry offer a wide range of possibility of design and development of exciting organophosphorus flame retardants. Conventional synthetic methodologies Michael addition, Michaelis–Arbuzov, Friedels–Crafts and Grignard reactions are the most commonly used in academics for development of P-C bonded flame retardants. Use of green reagents, catalytic reactions, microwave technology, photo initiated reactions, etc. are increasingly used by researchers to synthesize P-C containing organophosphorus compounds; however, these techniques are seldom utilized in development of flame retardant compounds. Though economics for these new routes for synthesis is important for future industrial exploitation, concerns for the environment and new regulations will help their acceptance in industrial exploitation. In our opinion, some synthetic strategies like Michael addition, Friedel–Crafts reaction and Michaelis–Arbuzov reaction can be industrially exploited in industry, taking in their advantages like higher yields, selectivity and simplicity of reaction. However, other reactions discussed in this review are also commercially feasible if the properties of the flame retardant significantly outweigh its cost of production.

One can find a vast amount of literature in development of P-C containing flame retardants with detailed investigations of their flame retardant properties and potential applications in various polymers. In most cases, such flame retardants exhibit satisfactory to excellent fire performance in the polymers, and questions regarding their impact on their processability, mechanical performance, and long-term serviceability of the final products need more attention. As organophosphorus compounds are also toxic (used as pesticides, herbicides, insecticides, nerve gasses), one has to pay attention while developing new structures. Toxicity screening of the new compounds should be carried out early in their development to prevent accidents and potential issues relate to toxicity. Research on ways to hydrolyze P-C bonds will help reduce their potential environmental impact and biocompatibility.

## Figures and Tables

**Table 1:** P-H transformation into P-C bond.

Reaction Type	General Examples	Selected Ref.
Pudovik reaction	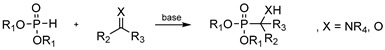	[6,30,37,38,39]
Kabachnik–Fields reaction	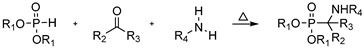	[6,30]
Michaelis–Becker reaction	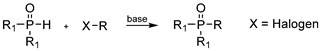	[26]
Nucleophilic ring opening of epoxides	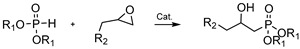	[6]
C-O/P-H cross coupling reaction	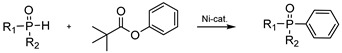	[40]
Dehydrogenative coupling with terminal alkynes	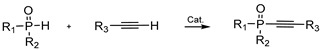	[6,12,41]
P-H insertion of α-Imino-carbenes	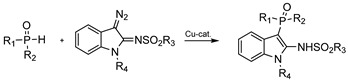	[42]
O_2_-induced radical oxyphosphorylation reaction	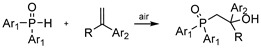	[43]
	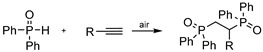	[44]
Phospha–Michael addition reaction	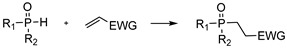	[5,15,29]

**Table 2:** Rearrangement/transformation Reactions.

Reaction Type	General Examples	Selected Ref.
Michaelis–Arbuzov reaction	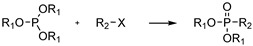	[5,27,28,45,46]
Sandmeyer-type reaction	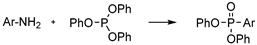	[47,48]
Decarbonylation of Acylphosphines	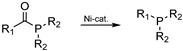	[49]
Abramov reaction	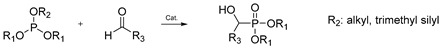	[30,35,50]

**Table 3:** C-H to C-P transformation.

Reaction Type	General Examples	Selected Ref.
Friedel–Crafts reaction	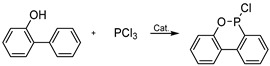	[5,8]
Via Aryne formation	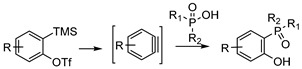	[51]
Nucleophilic substitution	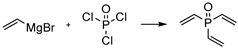	[8,52]

## Abbreviations

**ABS**	Acrylonitril butadiene styrene
**AOPH**	Aluminium–organophosphorus hybrids
**BKZ-VB**	Swiss standart fire test (vertical specimen)
**CPPPA**	4-Carboxyphenylphenylphosphinic acid
**DBU**	1,8-Diazabicyclo(5.4.0)undec-7-ene
**DDM**	Diaminodiphenylmethane
**DETDA**	Diethyldiamino toluene
**DFDPS**	4,4-Difluorodiphenylsulfone
**DGEBA**	Diglycidyl ether of bisphenol-A epoxy resin
**DIP-MS**	Direct insertion probe mass spectroscopy
**DOPO**	10-Dihydro-9-oxa-10-phosphaphenanthrene 10-oxide
**DPPO**	2,8-Dimethyl-phenoxaphosphine-10-oxide
**DSC**	Differential scanning calorimetry
**EDX**	Energy-dispersive X-ray spectroscopy
**EVA**	Ethylene-vinyl acetate
**FPUF**	Flexible polyurethane foams
**FR**	Flame retardant
**FTIR**	Fourier Transform Infrared Spectroscopy
**HAPP**	Hexaallyltriphosphazene
**KMnO4**	Potassium permanganate
**LOI**	Limiting oxygen index
**MCC**	Microscale Combustion Calorimetry
**m-CPBA**	meta-Chloroperoxybenzoic acid
**MMPP**	Methyl methylphenylphosphinate
**m.p.**	Melting point
**N_2_**	Nitrogen
**PA6**	Polyamide 6
**PC**	Polycarbonate
**PCFC**	Pyrolysis-combustion flow calorimetry
**PEEK**	Polyether ether ketone
**PEK**	Polyether ketones
**PES**	Poly(ethersulfone)
**PHRR**	Lower peak heat release rate
**PMMD**	Poly(ethylene glycol) methacrylate/methyl methacrylate/diethyl allylphosphonate
**PN-TLCP**	Thermotropic liquid crystal polymer
**POSS**	Polyhedral oligomeric silsesquioxane
**PSU**	Polysulfones
**PU**	Polyurethane
**Py-GC-MS**	Pyrolysis-gas chromatography mass spectroscopy
**ROMP**	Ring opening metathesis polymerisation
**RPUF**	Rigid polyurethane foams
**SEM**	Scanning electron microscope
**SET**	Self-extinguishing time
**TCPP**	Tris(1-chloro-2-propyl) phosphate
**Tdx%**	Temperature for x% weight loss
**TEOS**	Tetraethyl orthosilicate
**TGA**	Thermogravimetric analysis
**THR**	Total heat release
**TPA**	Terephthalic acid
**TSR**	Total smoke release
**UL94 HB**	Standard for Tests for Flammability of Plastic Materials for Parts in Devices and Appliances (horizontal specimen)
**UL94 VA or UL94 VB**	Standard for Tests for Flammability of Plastic Materials for Parts in Devices and Appliances (vertical specimen)
**UV**	Ultraviolet
**WAXD**	Wide-angle X-ray scattering
**XPS**	X-ray photoelectron spectroscopy
